# Predictors of one-year mortality after hospitalization for an exacerbation of COPD

**DOI:** 10.1186/s12890-018-0574-z

**Published:** 2018-01-25

**Authors:** Cristóbal Esteban, Ady Castro-Acosta, Carlos Jose Alvarez-Martínez, Alberto Capelastegui, José Luis López-Campos, Francisco Pozo-Rodriguez

**Affiliations:** 1 Pulmonary Service, Galdakao-Usansolo Hospital, Galdakao, Bizkaia Spain; 2 Pulmonary Service and Research Institute, Doce de Octubre University Hospital, Madrid, Spain; 3 CIBER Enfermedades Respiratorias (CIBERES), Madrid, Spain; 4 Hospital Universitario Virgen del Rocío, Medical- Surgical Unit of Respiratory Diseases, Seville, Spain; 5 Network and Health Services Research Chronic Diseases (REDISSEC), Bilbao, Bizkaia Spain

**Keywords:** Pulmonary disease, Chronic obstructive, Hospitalization, Mortality

## Abstract

**Background:**

Hospitalization for a severe exacerbation of COPD (eCOPD) is an important event in the natural history of COPD. Identifying factors related to mortality 1 year after hospitalization could help determine interventions to reduce mortality.

**Methods:**

In a prospective, observational, multicentre study, we evaluated data from two cohorts: the Spanish audit of hospital COPD exacerbation care (our derivation sample) and the Spanish cohort of the European audit of COPD exacerbation care (our validation sample). The endpoint was all-cause mortality. Mortality was determined by local research managers of the participating hospitals and matched the official national index records in Spain.

**Results:**

In the multivariate analysis, factors independently related to an increase in mortality were older age, cardio-cerebro-vascular and/or dementia comorbidities, PaCO2 > 55 mmHg measured at emergency department arrival, hospitalizations for COPD exacerbations in the previous year, and hospital characteristics. The area under the receiver-operating curve for this model was 0.75 in the derivation cohort and 0.76 in the validation cohort.

**Conclusion:**

One-year mortality following the index hospitalization for an exacerbation of COPD was related to clinical characteristics of the patient and of the index event, previous events of similar severity, and characteristics of the hospital where the patient was treated.

## Background

Hospitalization for a severe exacerbation of chronic obstructive pulmonary disease (eCOPD) is an important event in the natural history of this disease. Approximately half of the patients admitted for an eCOPD die within 4 years [1]. Identifying factors associated with mid-term mortality (1 year) after hospitalization for an eCOPD could provide tools for more targeted evaluation of these patients and interventions to prevent premature death.

Several studies designed for that purpose have yielded variable or conflicting results [2, 3]. These divergent results can largely be attributed to study methodology, such as the patient population (recruited from the medical ward or from intermediate or intensive care), source of the data (prospective studies, retrospective studies, or computerized databases), variables studied, duration of follow-up, and organizational and administrative distribution of hospital resources. Another source of uncertainty is whether indices used among patients with stable COPD have the same prognostic capacity when used in those admitted to the hospital [4, 5].

The aim of this study was to determine predictors of one-year mortality in patients hospitalized for an eCOPD following discharge for the index event.

## Methods

The Cohorte Española de EPOC Avanzada (CEPA) is a prospective observational study designed to examine factors associated with one-year mortality among patients hospitalized for an eCOPD.

CEPA includes patients from two separate cohorts. One, defined as the derivation cohort, is a sample of patients from the Spanish COPD Audit [6]. The other, defined as the validation cohort, is from the Spanish sample of the European COPD Audit [7]. The methodology of both studies has been described in detail elsewhere [6, 7].

The Spanish COPD Audit was a cross-sectional study with prospective non-interventional case ascertainment of consecutive eCOPD hospital admissions for the 8-week period from November 1, 2008 to December 31, 2008. Patients were included in the cohort following a two-step process. Reports of all patients admitted to the hospital following an emergency department (ED) visit were reviewed daily to identify one or more of 13 clinical conditions compatible with the diagnosis of eCOPD. These patients were labelled as interim/possible eCOPD cases. They were assessed again at hospital discharge against a list of definite inclusion and exclusion criteria to identify patients with a clinical diagnosis of eCOPD. Those who qualified were labelled as definite eCOPD cases [6]. A total of 32 hospitals completed a one-year follow-up of their patients and were included in our study.

The European Respiratory Society COPD audit was designed as a pilot study to evaluate clinical practice variability as well as clinical and organizational factors associated with outcomes for COPD-related hospital admissions across Europe. This prospective, observational, non-interventional cohort trial was conducted during the 8-week period from January 1, 2011 and February 28, 2011 in 74 hospitals [7].

In both studies, all consecutive cases admitted to the participant hospitals due to an eCOPD were identified and information on clinical practice was gathered. Each patient was included only once in the study.

Comorbidities were standardized recorded from the clinical record of every patient, including any new one diagnosed during the index event. The Charlson index was used as a reference [8].

All the hospitals participating in the studies were asked to complete cross-sectional surveys of the resources and organization they devoted to COPD acute care. The survey included questions related both to the hospital and to the respiratory unit resources.

Mortality was determined by local research managers of the participating hospitals and matched the official national index records in Spain.

The study was approved by Instituto de Salud Carlos III CEI PI 30_2011-v4 and hospital participation in the two cohorts was approved by the ethics committees of the participating hospitals. All participants provided written informed consent. To allow for patient-level analyses, patient data was protected and dissociated.

### Statistical analysis

Descriptive statistics of sociodemographic and clinical variables were calculated using means and standard deviations (SD) for quantitative data; frequencies and percentages were used for categorical variables. The Spanish cohort represented our derivation cohort and the European cohort the validation cohort.

A univariate analysis was undertaken in the derivation sample to determine variables that were predictors of one-year mortality (patients who died during the admission were excluded from the analysis). Student’s t test (or the Wilcoxon test if normality was rejected) was used for continuous variables, while Chi-square and Fisher’s exact tests were used for categorical variables. In the multivariate analysis, generalized linear mixed models were used so as to perform a multilevel multivariable analysis adjusted by hospital, with one-year mortality as the dependent dichotomous variable. The predictive accuracy of the model was determined by calculating the area under the receiver-operating curve (AUC).

The model developed in the derivation cohort was tested in the validation cohort to compare the predictive accuracy of the model by means of comparisons of their AUCs.

We also evaluated the independent influence of the hospital setting both models and determined which hospital characteristics were predictors of mortality.

All effects were deemed statistically significant at *p* < 0.05. All statistical analyses were performed using SAS System, version 9.2 (SAS Institute, Inc., Carey, NC).

## Results

A sample of 1420 patients from the Spanish COPD Audit was included in the derivation cohort. Mean patient age was 73 years, 89% were men, 24.6% were current smokers, 43% had a Charlson Comorbidity Index higher than 3, and 74.6% had previously been admitted for an eCOPD (Table 1). The in-hospital mortality for that cohort was 5%.

**Table 1:** Descriptive statistics of the COPD derivation and validation cohorts

	Derivation *n* = 1420	Validation *n* = 3949	*p*-value
Age^a^	73.4 (10.0)	72.7 (10.7)	0.07
Sex (% male)	1275 (89.8)	3375 (85.5)	< 0.0001
FEV1%	45.2 (16.2)	45.3 (16.6)	0.76
FEV1%/FVC	52.5 (13.4)	52.5 (12.9)	0.79
FEV1% categories			< 0.0001
≥ 80%	33 (3.8)	62 (2.5)	
< 80 - > 50	272 (31.2)	778 (31.0)	
< 50 - > 30	217 (24.3)	1187 (47.3)	
≤ 30	351 (40.2)	482 (19.2)	
Body Mass Index^a^	27.9 (5.3)	27.8 (5.7)	0.35
Smoking habit			0.34
- Ever smoker	52 (4.3)	182 (4.9)	
- Current smoker	343 (28.2)	977 (26.3)	
- Ex-smoker	822 (67.5)	2554 (68.8)	
Cigarette pack-years^a^	55,82 (29.0)	51,99 (30.3)	0.003
Charlson Comorbidity Index^a^	2,70 (1.8)	1,79 (1.7)	< 0.0001
1–2	795 (56.0)	2865 (72.5)	
≥ 3	625 (44.0)	1084 (27.4)	
Ischaemic heart disease	205 (15.7)	505 (12.8)	0.008
Cardiac failure	299 (23.0)	810 (20.5)	0.07
Peripheral vascular disease	221 (17.2)	494 (12.5)	< 0.0001
Cerebrovascular disease	146 (11.3)	380 (9.6)	0.09
Cardiac-Cerebro-Peripheral vascular disease	608 (46.2)	2217 (44.9)	0.41
Dementia	138 (3,49)	52 (4,04)	0.36
Diabetes mellitus	359 (26.2)	1032 (26.1)	0.95
Previous COPD hospitalization	1065 (76.7)	1881 (51.3)	< 0.0001
Mean of COPD-related admissions in the previous year	1.92 (2.0)	1.17 (1.8)	< 0.0001
pH at arrival	7.40 (0.1)	7.40 (0.1)	0.12
Acidaemia during admission	258 (19.3)	NA	
PaO2 at arrival^a^	59.1 (15.6)	60.4 (21.3)	0.79
PaCO2 at arrival^a^	47.2 (14.4)	48.4 (15.7)	0.074
Length of stay^a^	10.1 (6.9)	9.3 (7.0)	< 0.0001

^a^Represented as mean (std)

The validation cohort included a sample of 3949 patients from the European COPD Audit. Compared to the derivation cohort, the validation cohort included more women (10.2 vs 14.5%), had a lower Charlson Comorbidity Index, and statistically significant differences were observed in the percentages of cardiovascular, cerebrovascular, and peripheral vascular disease between the two cohorts (Table 1). The in-hospital mortality for that cohort was 4.3%.

In the univariate analysis, statistically significant differences were observed between patients who had died within 1 year of the index hospitalization and those who had not, in age, smoking habit, Charlson Comorbidity Index, and presence of specific comorbidities such as cardiovascular disease, heart failure, peripheral vascular disease, cerebrovascular disease and dementia, previous eCOPD admissions, number of eCOPD admissions in the previous year, level of airway severity obstruction (FEV1% and Global Initiative for Chronic Obstructive Lung Disease [GOLD] stages of obstruction) and PaCO2 level upon ED arrival. At discharge, patients who died within 1 year were more likely to have been prescribed home oxygen therapy than those who did not die, while prescription of home mechanical ventilation support at discharge and length of stay were not different between these groups (Table 2).

**Table 2:** Univariate analysis performed in the derivation sample (*n* = 1420)

	Mortality		
	No 864	Yes 495	*p*-value
Age^a^	71.7 (10.2)	76.1 (9.1)	< 0.0001
Sex (% male)	763 (88.3)	453 (91.5)	0.06
FEV1%	46.6 (16.3)	43.2 (15.4)	0.004
FEV1%/FVC	53,0 (12.9)	51.6 (14.2)	0.18
FEV1% categories			< 0.0001
≥ 80%	24 (4.5)	7 (2.2)	
< 80 - > 50	183 (34.0)	85 (27.3)	
< 50 - > 30	144 (26.8)	62 (19.9)	
≤ 30	187 (34.8)	157 (50.5)	
Body Mass Index^a^	28.1 (5.3)	27.3 (5.4)	0.07
Smoking habit			0.0007
- Never smoker	29 (3.8)	19 (4.7)	
- Current smoker	246 (32.4)	89 (21.8)	
- Ex-smoker	485 (63.8)	300 (73.5)	
Cigarette pack-years^a^	53.2 (27.3)	60.9 (31.9)	0.002
Charlson Comorbidity Index^a^	2.5 (1.6)	3.1 (1.9)	< 0.0001
1–2	536 (62.0)	234 (47.3)	
≥ 3	328 (38.0)	261 (52.7)	
Ischaemic heart disease	111 (14.0)	84 (18.3)	0.04
Cardiac failure	148 (18.7)	129 (28.3)	< 0.0001
Peripheral arterial disease	113 (14.4)	94 (20.8)	0.004
Cerebrovascular disease	67 (8.5)	72 (15.9)	< 0.0001
Cardiac-Cerebro- Peripheral vascular disease	320 (40.1)	250 (54.2)	< 0.0001
Dementia	17 (2.18)	31 (6.84)	< 0.0001
Diabetes mellitus	208 (25.1)	129 (26.9)	0.46
Previous COPD hospitalization	610 (71.9)	404 (83.8)	< 0.0001
Mean of COPD-related admissions in the previous year	1.7 (1.9)	2.2 (2.2)	0.001
pH at arrival	7.40 (0.06)	7.40 (0.06)	0.49
Acidaemia during admission	150 (18.4)	84 (18.1)	0.89
PaO2 at arrival^a^	58.8 (15.0)	59.6 (16.2)	0.53
PaCO2 at arrival^a^	46.2 (13.0)	48.5 (15.2)	0.01
Length of stay^a^	10.4 (7.2)	9.7 (6.4)	0.11
Oxygen at discharge	329 (38.1)	276 (55.8)	< 0.0001
Noninvasive ventilation at discharge	48 (5.6)	29 (5.9)	0.81

^a^Represented as mean (std)

In the multivariate analysis, independent variables related to one-year mortality were age, comorbidity (making reference to previous presence of cardiac, peripheral vascular or cerebral vascular diseases and dementia), admission for an eCOPD in the previous year, and PaCO2 at emergency department arrival, and the participant hospital. The hospital characteristics that influenced one-year mortality were the size of the hospital and whether they had a respiratory ward (Table 3).

**Table 3:** Univariate analysis. Hospital characteristics

	Dead *N* (%)	Alive *N* (%)	*p*-value
Hospital			< 0.0001
Total bed numbers^a^	601.0 (359.3)	627.3 (312.0)	0.005
Total bed numbers			< 0.0001
- < 300	115 (23.2)	134 (15.5)	
- 300–599	188 (38.0)	294 (34.0)	
- ≥ 600	192 (38.8)	436 (50.5)	
Population served by the hospital			0.01
- < 200,000	199 (40.2)	278 (32.2)	
- 200,000–299,999	167 (33.7)	338 (39.1)	
- ≥ 300,000	129 (26.1)	248 (28.7)	
Teaching Hospital			0.60
- No	241 (48.7)	407 (47.1)	
- Yes	254 (51.3)	457 (52.9)	
Intensive Care Unit			0.17
- No	30 (6.1)	38 (4.4)	
- Yes	465 (93.9)	826 (95.6)	
Spirometry available			0.47
- No	56 (11.3)	87 (10.1)	
- Yes	439 (88.7)	777 (89.9)	
Respiratory staff number^a^	9.43 (6.2)	9.49 (5.8)	0.44
Respiratory ward			0.04
- No	91 (18.4)	123 (14.2)	
- Yes	404 (81.6)	741 (85.8)	
Non-invasive ventilation available in the ward			0.47
- No	26 (5.2)	38 (4.4)	
- Yes	469 (94.7)	826 (95.6)	

^a^Represented as mean (std). *N* frequency, *%* percentage

The AUC for this model was 0.75 for the derivation cohort and 0.76 for the validation cohort (Tables 4 and 5).

**Table 4:** Derivation cohort. Multivariate analysis. Predicting factors of mortality in 1 year

	β (s.e.)	OR (95% CI)	*p*-value	Weight	AUC
Intercept	− 2.12 (0.56)		0.0002		0.672
Age^b^	0.46 (0.08)	1.581 (1.357–1.842)	< 0.0001	2	
COPD related previous admissions (Yes vs. No)	0.79 (0.18)	2.201 (1.541–3.144)	< 0.0001	4	
Cardio-cerebro-peripheral vascular disease^a^ (Yes vs. No)	0.47 (0.15)	1.598 (1.189–2.148)	0.0019	2	
Dementia (Yes vs. No)	1.09 (0.39)	2.973 (1.394–6.340)	0.0048	5	
PaCO2					
45–55 (vs. < 45)	− 0.05 (0.18)	0.947 (0.663–1.353)	0.7631	0	
> 55 (vs. < 45)	0.47 (0.19)	1.601 (1.102–2.326)	0.0135	2	
Hospital characteristics					0.747

Hospital characteristics: defined by “total number of beds”

*β* parameter estimation, *s.e.* standard error, *OR* odds ratio, *CI* confidence interval, *AUC* area under the receiver operating characteristic Curve, *Weight* indicates the weight for each variable to create the risk score

^a^Include ischaemic heart disease, cardiac failure, peripheral vascular disease and cerebrovascular disease

^b^Estimation for increment of a decade with respect to patients with 50 years or less

*p*-value Hosmer y Lemeshow = 0.26 (including in the analysis hospital *p*-value = 0.19)

**Table 5:** Validation cohort. Multivariate analysis. Predicting factors of COPD mortality in 1 year

	β (s.e.)	OR (95% CI)	*p*-value	AUC
Intercept	− 3.08 (0.55)		< 0.0001	0.672
Age^b^	0.43 (0.06)	1.530 (1.367–1.712)	< 0.0001	
COPD related previous admissions (Yes vs. No)	0.72 (0.11)	2.045 (1.640–2.550)	< 0.0001	
Cardio-cerebro-peripheral vascular disease^a^ (Yes vs. No)	0.31 (0.11)	1.367 (1.097–1.705)	0.0054	
Dementia (Yes vs. No)	0.50 (0.26)	1.648 (0.984–2.758)	0.0575	
PaCO2				
45–55 (vs. < 45)	0.22 (0.13)	1.245 (0.965–1.607)	0.0917	
> 55 (vs. < 45)	0.51 (0.13)	1.665 (1.280–2.165)	0.0001	
Hospital characteristics				0.763

Hospital characteristics: defined by “total number of beds”

*β* parameter estimation, *s.e.* standard error, *OR* odds ratio, *CI* confidence interval, *AUC* area under the receiver operating characteristic curve

^a^Include ischaemic heart disease, cardiac failure, peripheral arterial disease and cerebrovascular disease

^b^Estimation for increment of a decade. With respect to patients with 50 years or less

*p*-value Hosmer y Lemeshow = 0.25 (including in the analysis hospital *p*-value = 0,37)

A score based on the weight of the different variables included in the multivariate model was developed (Table 4). This score was stratified in 4 levels that showed a high rate of mortality at every risk level, especially in those patients having a score higher than 10 (Table 6).

**Table 6:** Description of the mortality rate at 1 year in the different risk groups

	Derivation sample			Validation sample		
	*N* (%)	Mortality 1 year	*p*-value	*N* (%)	Mortality 1 year	*p*-value
Risk groups			< 0.0001			< 0.0001
Low (0–5)	83 (8.03)	7 (8.43)		498 (15.39)	17 (3.41)	
Low-Medium (6–7)	112 (10.83)	22 (19.64)		513 (15.86)	38 (7.41)	
Medium-High (8–10)	412 (39.85)	134 (32.52)		1307 (40.40)	201 (15.38)	
High (>10)	427 (41.30)	206 (48.24)		917 (28.35)	236 (25.74)	
AUC (95% CI)	0.740 (0.709–0.771)			0.763 (0.741–0.784)		

*N* frequency, *%* percentage, *CI* confidence Interval

## Discussion

In this prospective cohort study, four groups of variables were associated with one-year mortality after an eCOPD hospitalization: general condition of the patient (older age, presence of cardiovascular, cerebrovascular, or peripheral vascular disease and dementia); clinical markers of the severity of the index event, (PaCO2 above the normal limit, at ED arrival); previous events of similar severity (hospitalization for an eCOPD in the previous year). The presence of any of these variables, added to the characteristics of the hospital where the patient was treated, were associated with higher mortality.

### Patient’s characteristics

Age, as a continuous variable, was an independent predictor of mortality. Age has been included, although controversially, as a variable in other multidimensional indexes for stable COPD [9, 10]. Among patients experiencing severe exacerbations, age is a key predictive factor for in-hospital mortality [11] and long-term post-discharge mortality [12]. Data from 150 hospitals in the United Kingdom indicated that age was the main predictor of 30-day mortality following hospitalization for COPD (OR, 8.5; 95% CI, 7.7–9.4). Other predictive factors included comorbidities, and gender [13]. In other studies, age was an independent predictor of mortality during one-year of follow-up after hospitalization for an eCOPD [14, 15]. It must be noted, however, that age is a stronger predictor of long-term mortality following eCOPD hospitalization than it is as a predictor for in-hospital mortality [16].

Several reasons could explain our previously mentioned results, older COPD patients tend to have a low performance status, higher level of respiratory disability, and more comorbidities. Together with a diminished awareness of the signs of an exacerbation and poorer understanding of medication [17], these factors make older patients extremely vulnerable, with generally worse outcomes and increasing 90-day mortality [17]. Moreover, respiratory specialists usually find it more difficult to control COPD in older patients than in younger ones [17]. In the UK audit, hospital care varied with age, but the lower level of care given to older patients was not associated with mortality [18]. In sum, all of these circumstances could make age a key factor contributing to mortality after hospitalization for an eCOPD.

Although comorbidities are prevalent among hospitalized eCOPD patients [19], there is some controversy over their importance as a prognostic factor for mortality. In one study, non-respiratory organ failure was most closely related to in-hospital mortality while the overall level of respiratory severity was associated with long-term mortality [16].

In another study comorbidities—cor pulmonale, left ventricular failure, neurological conditions, and non-respiratory malignancies—were associated with in-hospital mortality while 90-day mortality was also increased by the presence of lung cancer or arrhythmias [19]. Almagro et al. showed that the Charlson Comorbidity Index score were associated with 90-day mortality [20]. Comorbidities such as cardiac failure [21], ischaemic heart disease [22], stroke, and diabetes [23] have also been identified as predictors of mortality after hospitalization for an eCOPD.

We did not observe an association between comorbidities evaluated by the Charlson index and mortality during the one-year follow-up, as in another study was [24]. In contrast, in our cohort specific comorbidities—cardiac disease (ischaemic heart disease, chronic heart failure), cerebrovascular disease, and peripheral vascular disease—were independently related to mortality. It seems that the impact of the different comorbidities is variable with respect to the outcome analysed [25] and is focused on the cardio-cerebro-vascular comorbidities, because not all the comorbidities have the same importance.

Apart from the cardio-cerebro-vascular comorbidities, it is worth mentioning dementia as a variable associated to mortality in our study. Prevalence of cognitive dysfunction is very variable in COPD patients, (up to 61%), depending on the place where the study was carried out [26].

COPD patients are prone to being affected by deterioration of their level of cognitive function, and there are several mechanisms involved, from the hypoxaemia, usually present in COPD, to smoking and some concurrent comorbidities, as cardiovascular [27].

Cognitive deterioration has been shown to have a great impact on important outcomes. In patients admitted to a general hospital those with dementia had a mortality rate of 48% after 1 year, higher than that of patients without dementia [28]. In COPD, those patients admitted suffering dementia had 38% and 69% higher risk of severe sepsis and hospital mortality respectively [29].

Our study adds that in COPD patients, dementia was a key point and the strongest variable associated to mortality in the mid term, after one hospitalization.

In sum, what our study showed was the important relationship between comorbidities and mortality, but not comorbidities in general, but some specific comorbidities such as cardio-cerebro-vascular and dementia.

### Factors of the index event (hospitalization)

Several variables were registered from the hospitalization, however our study showed that only hypercapnia at the time of hospital admission was associated with one-year mortality.

PaCO2 has been recognized as a key predictor of in-hospital mortality [30] and one-year after admission mortality [14, 22] among eCOPD patients. A study that included COPD patients admitted to the intensive care unit established that the severity of the exacerbation reflected by respiratory physiology influenced one-year mortality but not in-hospital mortality [16]. Patients admitted with a PaCO2 > 50 mmHg—which would indicate the severity of the underlying respiratory disease, like ventilation/ perfusion mismatch or hypoventilation—had higher one-year mortality than those with PaCO2 < 50 mmHg. It is possible that in this study, the homogeneity of the cohort (ICU patients only) could have diluted the discriminative capacity of the analysis, limiting the possibility of finding any relationship between in-hospital mortality and variables (like PaCO2) that reflect the physiological respiratory impairment during an eCOPD.

We were not able to demonstrate if hypercapnia persisted at discharge, and if its persistence was related to mortality, as has been suggested by others [20]. Neither did we have any information on patient PaCO2 in a stable condition of our cohorts.

### Previous hospitalizations

Previous eCOPD hospitalizations have been identified as an independent predictor of mortality in cohorts of stable and hospitalized patients. Soler-Cataluña et al., for example, demonstrated that mortality increases with the frequency of severe exacerbations requiring hospital evaluation [31]. Other researchers have observed that the number of severe eCOPD requiring hospitalization increases the risk of respiratory and all-cause mortality regardless of factors such as age, FEV_1_, dyspnoea, cumulative pack-year smoking history, and health-related quality of life [32].

Across the lifespan of COPD patients, the addition of every new hospitalization significantly increases the rate of mortality, and the risk of other severe eCOPD peaks during the 3 months after the index event [1].

In cohorts derived from databases, episodes of previous COPD-related hospitalizations were an independent predictor of mortality [21, 33]. In fact, previous hospitalizations and comorbidities have been included as components of a multidimensional score predicting mortality in a cohort of hospitalized patients.

In our study having had one hospitalization the year before the index-event increased the probability of mortality the year after by 60%, which would be in accordance with the results of studies previously mentioned and outline more precisely the profile of the patient that should be carefully monitored after and eCOPD hospitalization.

### Hospital characteristics

Hospital characteristics were reported as a predictor of mortality in the UK national audit 2003. Inpatient and 90-day mortality were lower in hospitals with more respiratory staff per 1000 beds. No other associations were found between other hospital resources and mortality.

The European COPD audit team evaluated adherence to 10 key management recommendations set forth by the GOLD standards in 13 European countries. It found great variability between hospitals and across countries, especially connected with the availability of the level of severity of the obstruction and the use of oxygen and non-invasive ventilation. The audit also showed that larger hospitals have more resources but this does not imply a better quality of care.

Walker et al. showed that 30-day mortality after hospitalization for an eCOPD was quite variable from 1 year to the following across various hospitals. Mortality in that period was independent of hospital size and of whether a respiratory specialist was responsible for the discharge of the patient [13].

In our study, hospital size, as measured by bed numbers and population served, was related to one-year mortality. Knowing the potential limitation of using data from one brief time point rather than data collected over several years we could speculate that having more resources provided not only better treatment of the index episode but better follow-up care afterward. We observed a significant influence of the hospital on one-year mortality since the AUC increases from 0.68 to 0.77 after including the variable hospital in the analysis. Nevertheless, we were not able to establish whether this is due exclusively to the hospital, or is also due to the primary care organization, the regional medical or social characteristics involved in the care of these patients, or all of these together. In any case including hospital size seems to be an important point in the final results.

### Strengths and limitations

Strengths of our study included are the number of patients we were able to follow, the length of follow-up, and the fact that patients were recruited from multiple centres. The usage of multilevel models provides a better approach than ordinary logistic regression models when patients are clustered in different groups, as in our case, that we have patients clustered in different hospitals*.* In this way, the correlation effect among patients from the same hospitals is controlled, as we do not consider them independently.

Several limitations must also be noted, primarily related to the design of the study as an audit of care in eCOPD, which implies missing data. We used for the analysis only those variables with less than 15% of missing data. Another limitation is that we were not able to recover the whole cohort of the Spanish audit. Instead, we analysed only those hospitals that conducted a follow-up of 1 year. We checked our sample against the whole cohort and verified that there were no differences in the clinical characteristics of the patients. Patients who died during the admission were not included in this analysis, only discharged patients. We were not able to pinpoint the cause of death because it is not accurately registered in death records, but this was not the objective of this study. As an audit of general practice some variables currently used as part of predictive mortality scores were not collected. Thus, a comparison with others mortality scores was not possible. The low percentage of women in both cohorts of the study limited the possibility of carrying out an analysis about potential differences with respect to men in the main outcomes studied.

## Conclusions

In a cohort of patients hospitalized for an eCOPD, predictors of mortality in the year following the index hospitalization were patient characteristic age, characteristics of the disease (admission for an eCOPD in the previous year and specific comorbidities), aspects of the index event (PaCO2), and the characteristics of the hospitals in which the patients were treated. Some of these aspect are potentially adjustable to impact in the care and outcomes of severe eCOPD.

In conclusion our study provides the patient profile with the highest probability of mortality during the year after an eCOPD hospitalization. We should pay attention to a man in his seventies, with cardio-vascular and/or dementia, with a previous eCOPD hospitalization the year before the index event and with hypercapnia > 50 mmHg during the admission. In those patients with these characteristics a more personalized follow-up is mandatory.

## Abbreviations

AUC: Area under the receiver-operating curve
COPD: Chronic obstructive pulmonary disease
eCOPD: Exacerbation of COPD
ED: Emergency department
